# Characteristics of the nocturnal desaturation waveform pattern of SpO_2_ in COPD patients: an observational study

**DOI:** 10.1186/s12931-021-01868-9

**Published:** 2021-10-26

**Authors:** Asuka Yoshizaki, Tatsuya Nagano, Shintaro Izumi, Teruaki Nishiuma, Kyosuke Nakata, Masatsugu Yamamoto, Yuichiro Yasuda, Daisuke Hazama, Kanoko Umezawa, Naoko Katsurada, Motoko Tachihara, Yoshihiro Nishimura, Kazuyuki Kobayashi

**Affiliations:** 1 Division of Respiratory Medicine, Department of Internal Medicine, Kobe University Graduate School of Medicine, 7-5-1 Kusunoki-cho, Chuo-ku, Kobe, Hyogo 650-0017 Japan; 2 Graduate School of System Informatics, Kobe University, 1-1-Rokkodai-cho, Nada-ku, Kobe, Hyogo 657-8501 Japan; 3 Department of Respiratory Medicine, Kakogawa Central City Hospital, 439 Honmachi, Kakogawa-cho, Kakogawa, Hyogo 675-8611 Japan; 4 Department of Respiratory Medicine, Steel Memorial Hirohata Hospital, 3-1, Yumesaki-cho, Hirohata-ku, Himeji, Hyogo 671-1122 Japan

**Keywords:** COPD, Nocturnal desaturation, Sustained pattern, Periodic pattern, Intermittent pattern

## Abstract

**Background:**

Nocturnal desaturation is common in patients with chronic obstructive pulmonary disease (COPD) and impacts disease exacerbation and prognosis. In our previous study, we developed a diagnostic algorithm to classify nocturnal desaturation from SpO_2_ waveform patterns based on data from patients receiving home oxygen therapy. In this study, we aimed to investigate nocturnal desaturation in patients with COPD based on SpO_2_ waveform patterns and the associations between the waveforms and clinical data.

**Methods:**

We investigated patients diagnosed with COPD and measured SpO_2_ and nasal airflow with a type 4 portable long-term recordable pulse oximeter. Then, we classified the SpO_2_ waveforms with the algorithm and compared the clinical data.

**Results:**

One hundred fifty-three patients (136 male and 17 female) were analysed. One hundred twenty-eight of the 153 (83.7%) patients had nocturnal desaturation, with an intermittent pattern (70.6%), sustained pattern (13.1%) and periodic pattern (68.0%). Intriguingly, desaturation with an intermittent pattern was associated with the apnoea-hypopnea index obtained with the portable monitor, and desaturation with a sustained pattern was associated with the cumulative percentage of time at a SpO_2_ below 90%.

**Conclusions:**

We found that nocturnal desaturation was frequently observed in patients with COPD and could be classified into 3 types of waveform patterns.

## Background

Chronic obstructive pulmonary disease (COPD) is a highly prevalent disease worldwide, and sleep-related desaturation in COPD is well known [1, 2]. Even patients with mild to moderate COPD have lower total sleep duration, lower sleep efficacy and worse sleep quality due to insomnia and awakenings at night, leading to a lower quality of life [3]. As COPD progresses, it has been reported that nocturnal desaturations in COPD contribute to the development of daytime respiratory failure, the frequency of acute exacerbations and the development of pulmonary hypertension and are associated with poor survival [4–6]. Although nocturnal desaturation may have an important impact on patients with COPD, less research has been focused on the waveform patterns of desaturation.

In our previous study, we analysed nocturnal SpO_2_ waveforms obtained from patients with chronic respiratory diseases receiving home oxygen therapy (HOT), and we showed that the SpO_2_ waveforms were very diverse [7]. Then, we found that each waveform was composed of a combination of three desaturation patterns: intermittent pattern, sustained pattern and periodic pattern. Moreover, we developed a diagnostic algorithm to automatically analyse these waveforms.

In clarifying the pathophysiology of desaturation, it is important to classify the desaturation pattern. Therefore, in the present study, we aimed to adapt the algorithm for patients with mild to moderate COPD who were not receiving oxygen therapy and analyse the association between the waveform patterns and the clinical features of COPD.

## Methods

### Patients

This study was performed between September 2017 and March 2020. The current study was conducted with the approval of the Ethics Committees or Institutional Review Board of Kobe University Hospital (permission number: 160208).

**Fig. 1:**
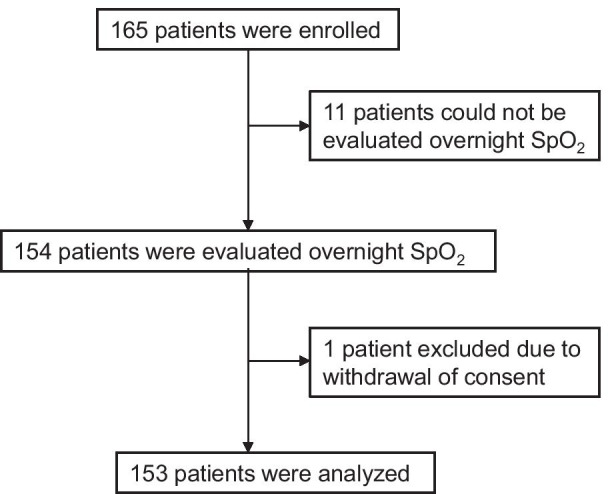
Recruitment of the patients

We enrolled 165 outpatients diagnosed with COPD who fulfilled the following inclusion criteria: aged 20 years or older; diagnosed with stable COPD; and daytime PaO_2_ ≥ 55 Torr (SpO_2_ ≥ 88%). COPD was diagnosed based on the Global Initiative for Chronic Obstructive Lung Disease (GOLD) guidelines (post-bronchodilator ratio of forced expiratory volume to forced vital capacity < 70%) [8]. Stable condition was defined as stable disease without reported exacerbations during the previous 3 months without any changes in respiratory medications. Patients were excluded from this study if they were receiving HOT (including positive-pressure ventilation therapy). Written informed consent was obtained from all study participants. The flowchart describing patient recruitment is shown in Fig. 1.

### Measurement and data collection

At registration, a pulmonary function test (PFT) was carried out. The PFT was performed by trained operators in accordance with the international recommendations [9, 10]. Japanese local reference values were used for the predicted values of the PFT [11]. During night, we monitored and evaluated the patients’ oxygenation and breathing with a portable long-term recordable pulse oximeter (SAS2100; Nihon Kohden, Corp., Tokyo, Japan). Recordings with duration of at least 4 h were accepted. Sex, body mass index, daytime SpO_2_ at rest, and smoking history were extracted from the medical records.

### Severity of COPD

Patients with COPD were classified as having GOLD 1 (forced expiratory volume in 1 s (FEV_1_) ≥ 80% predicted), GOLD 2 (50 ≤ FEV_1_ < 80% predicted), GOLD 3 (30 ≤ FEV_1_ < 50% predicted) and GOLD 4 (FEV_1_ < 30% predicted) disease based on the GOLD guidelines [8].

### Detection of desaturation

**Fig. 2:**
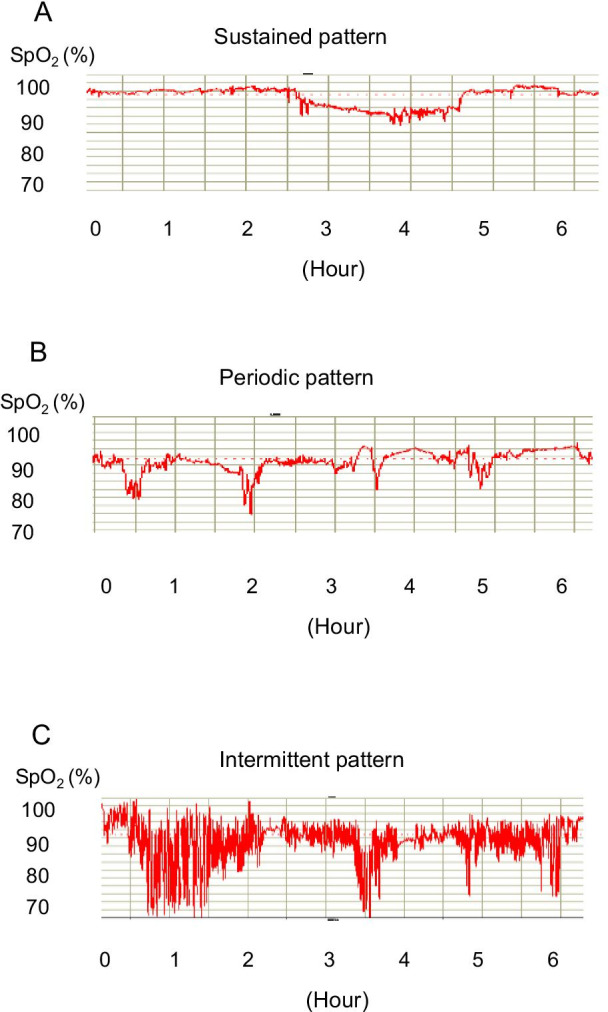
Characteristics of the nocturnal waveform pattern of SpO_2_ in COPD patients with nocturnal desaturation. **A** Sustained pattern, **B** periodic pattern and **C** intermittent pattern were classified based on the algorithm (Patients and Methods) ([12] (adapted from reference with permission)

In the present study, desaturation was defined as a more than 3% decrease in SpO_2_ from baseline. Nocturnal desaturations were divided into three patterns based on the algorithm that Izumi [12]. In brief, when desaturation events lasting longer than 655 s occurred, the events were labelled as the sustained pattern (Fig. 2A). Desaturation events between 30 and 655 s that occurred more than twice were labelled as the periodic pattern (Fig. 2B). We defined the third pattern as the intermittent pattern in which the decrease in and recovery of SpO_2_ was repeated with a cycle of several minutes (Fig. 2C). Finally, we defined the waveform with limited SpO_2_ changes other than those described above as the normal pattern.

SpO_2_ waveforms are diverse; however, we found that each waveform was composed of a combination of three desaturation patterns: S: Sustained, P: Periodic, and I: Intermittent. SpO_2_ waveforms can be classified into the following eight groups based on the combination of these three patterns: S, P, I, S + P + I, P + I, S + P, S + I and normal.

Apnoea was recorded as the cessation of airflow for at least 10 s, and hypopnea was defined as a ≥ 30% reduction in airflow for at least 10 s associated with an oxygen desaturation ≥ 3%. The apnoea-hypopnea index obtained from the portable monitor (PM-AHI) was defined as the number of apnoeas and hypopneas divided by the total time measured by the portable pulse oximeter. The cumulative percentage of the time spent at a SpO_2_ less than 90% (CT90) was calculated as the cumulative time spent at a SpO_2_ less than 90% divided by the total time measured by the portable pulse oximeter.

The 3% oxygen desaturation was defined as a more than 3% decrease in SpO_2_ from baseline and recover within 120 s. The 3% oxygen desaturation index (ODI) was defined as the number of 3% oxygen desaturations divided by the total time measured by the portable pulse oximeter.

The hypoxic burden was defined as the total area between the baseline and SpO_2_ waveform divided by the total time measured by the portable pulse oximeter, with the unit of hypoxic burden being (%min)/h [13].

### Statistical analysis

Comparisons concerning sex were performed using Fisher’s exact test. Comparisons between two groups other than sex were performed using the Mann–Whitney *U*-test. Comparison between seven groups was performed Kraskal-Wallis and Mann–Whitney *U*-test with Bonferroni’s correction. *P* values reported are 2-sided, and *P* values less than 0.05 were considered significant unless otherwise specified. *P* values after Bonferroni’s correction less than 0.0024 were considered significant.

## Results

### Patients and characteristics

One hundred fifty-three patients (136 male and 17 female) met the inclusion criteria. The median age of the participants was 72 years (interquartile range (IQR): 68–76), and their body mass index was 22.7 (IQR: 20.6–24.4). The numbers of patients with GOLD stages 1, 2, 3 and 4 were 27, 80, 38 and 4, respectively. These patients had a median daytime SpO_2_ of 97 (IQR: 96–98) %, and all participants had a daytime SpO_2_ ≥ 90%. The median percent predicted forced expiratory volume in 1 s (%FEV_1_) was 68.8 (IQR: 16.3–79.7), and the median lowest SpO_2_ was 85 (IQR: 80–87.5) %. Other patient characteristics, laboratory data and data from the portable oximeters are summarised in Table 1.

**Table 1:** Patients’ characteristics

	All N = 153	Desaturation + N = 128	Desaturation-N = 25	P value
Male/female	136/17	116/12	20/5	0.158
Age, years	72 [68–76]	71 [68–75]	73 [70.5–77.5]	0.063
BMI	22.7 [20.6–24.4]	22.7 [20.6–24.5]	22.6 [20.3–24.2]	0.542
Daytime SpO_2_, %	97 [96–98]	97 [96–98]	98 [96.3–98]	0.111
Smoking history, pack-years	50 [40–75]	50 [40–76.5]	45 [33.1–60]	0.115
Severity (Gold grade 1, 2, 3, 4)	27, 80, 38, 4 unknown 4	23, 68, 30, 3 unknown 4	4, 12, 8, 1	0.649
%FVC, %	89.7 [78.7–99.5]	90.1 [77.8–100.1]	85.8 [80.7–99.1]	0.932
%FEV_1_, %	59.8 [49.2–66.0]	62.4 [49.4–76.9]	63.3 [43.8–76]	0.576
FEV_1_/FVC, %	62.4 [47.9–76.7]	60.2 [50–66.1]	53.4 [40.5–63.3]	0.080
PM-AHI	15.2 [8.4–25.0]	17.2 [10.4–28.5]	6.6 [3.9–9.1]	< 0.0001*
CT90, %	1.48 [0.10–7.92]	2.7 [0.44–9.8]	0.03 [0–0.11]	< 0.0001*
Lowest SpO_2_, %	85 [79–89]	84 [78–87]	90 [88–91]	< 0.0001*

The data are presented as median [interquartile range]. *P < 0.05

PM-AHI, CT90% and Lowest SpO_2_ were obtained from a pulse oximeter performed during night

*BMI* body mass index, *FVC* forced vital capacity, *FEV*_*1*_ forced expiratory volume in 1 s, *PM-AHI* apnoea-hypopnea index from portable monitor, *CT90* cumulative percentage time at SpO_2_ below 90%

### Nocturnal desaturation

**Fig. 3:**
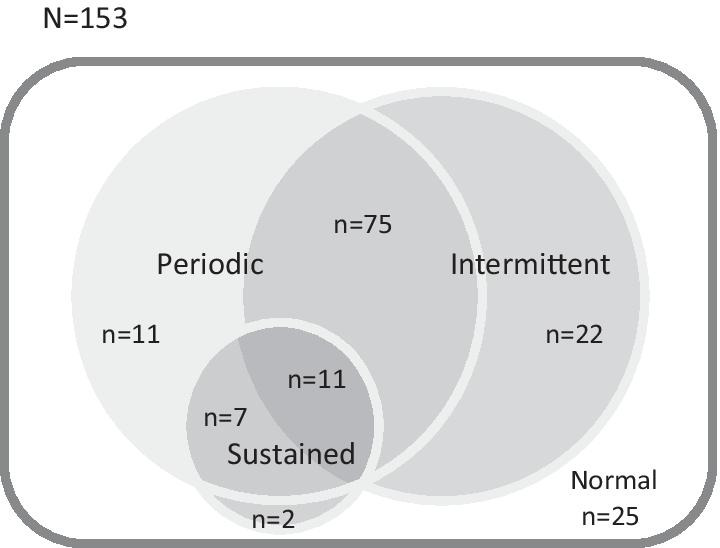
A Venn diagram of the waveform patterns

One hundred twenty-eight of the 153 (83.7%) patients had nocturnal desaturation. The determination of the results with the automatic analysis algorithm for the waveforms is shown below. Desaturation with the intermittent pattern was observed in 108 (70.6%), desaturation with the sustained pattern was observed in 20 (13.1%) and desaturation with the periodic pattern was observed in 104 (68.0%). Intriguingly, there was substantial overlap of the waveforms, especially the intermittent and periodic patterns. A Venn diagram for the waveform patterns is shown in Fig. 3.

### Associations between waveforms and clinical data

**Fig. 4:**
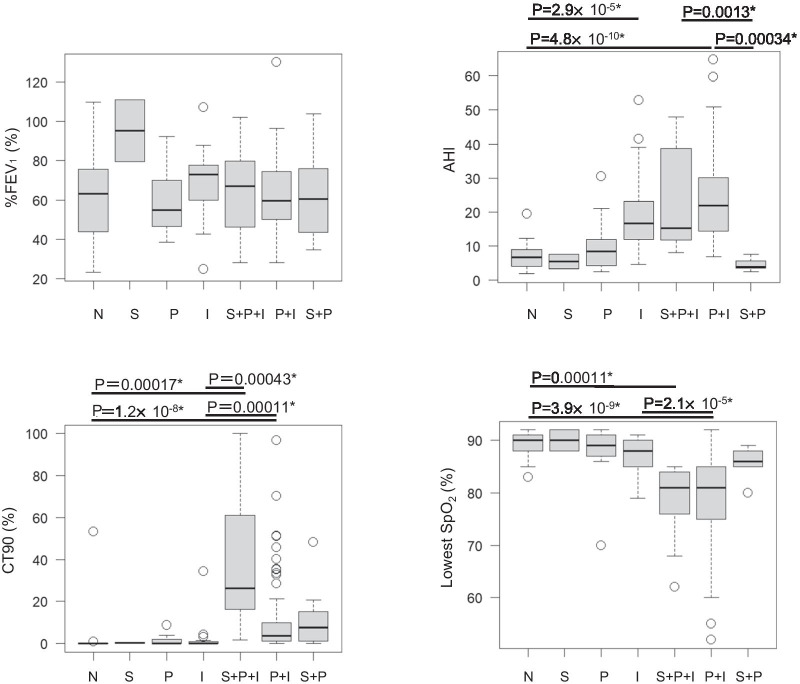
Association between the waveforms and the clinical data. *: Statistically significance with Bonferroni’s correction of multiple testing. N: Normal, S: Sustained, P: Periodic, I: Intermittent

**Fig. 5:**
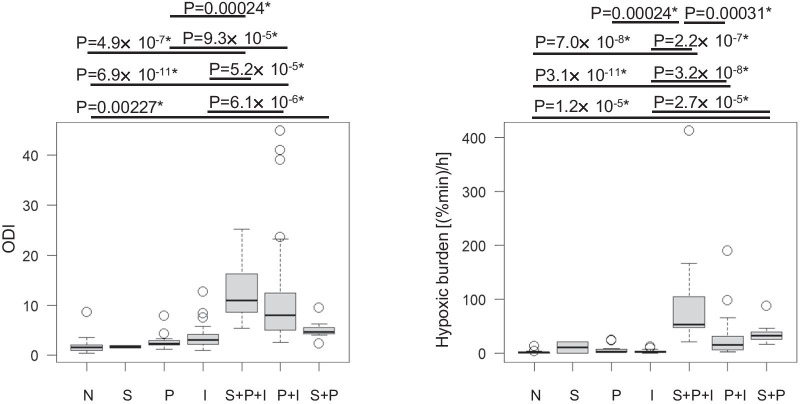
Association between the waveforms and desaturation parameters. *: Statistically significance with Bonferroni’s correction of multiple testing. N: Normal, S: Sustained, P: Periodic, I: Intermittent

We conducted an exploratory analysis to clarify the clinical data and desaturation parameters in each group. The results are shown in Figs. 4 and 5. In the groups with overlapping waveforms, especially the S + P + I group, the CT90 tended to be higher, and the lowest SpO_2_ tended to be lower (Fig. 4).

We assumed that the algorithm, which was developed based on data from patients with severe disease receiving HOT, was still indicative of the pathophysiological aspects in the current study population. Therefore, we compared the patients’ characteristics between the groups with or without each desaturation pattern.

The patients with the intermittent pattern had a significantly higher PM-AHI (19.5, IQR: 13.1–41.9 vs. 5.7, IQR: 3.5–9.3) and higher 3% ODI (7.4, IQR: 4.0–11.8 vs. 2.0, IQR: 1.3–3.3) than patients without the intermittent pattern (Table 2). And, in patients with intermittent pattern, 107 of 108 (99.1%) patients showed PM-AHI ≥ 5. The patients with high PM-AHI (≥ 15) were 75 (69.4%). These patients were relatively severe in the point of airway obstruction showing lower %FEV_1_, although not statistically significant (49.6, IQR: 28.1–75.8 vs 53.4, IQR: 24.9–76.6, P = 0.387). In addition, the hypoxic burden was significantly higher in patients with intermittent pattern than patients without intermittent pattern.

**Table 2:** Association of the intermittent pattern with clinical data

	Desaturation with intermittent pattern N = 108	Desaturation without intermittent pattern N = 20	P value
Male/female	98/10	18/2	1.00
Age, years	71 [68–75]	71.5 [67.3–75.8]	0.888
BMI	22.6 [20.3–24.6]	22.8 [21.2–24.3]	0.825
Daytime SpO_2,_ %	97 [96–98]	97 [96–97.8]	0.528
Smoking history, pack-years	50 [40–80]	60 [45.5–67.5]	0.737
%FVC, %	90.1 [78.0–99.2]	91.4 [76.6–102.7]	0.646
%FEV_1_, %	63.1 [50.6–76.6]	58.2 [46.0–79]	0.871
FEV_1_/FVC, %	60.2 [50–66]	60.3 [51.7–67.0]	0.970
PM-AHI	19.5 [13.1–41.9]	5.7 [3.5–9.3]	< 0.0001*
CT90, %	2.9 [0.72–13.2]	0.445 [0.025–6.7]	0.024*
Lowest SpO_2_, %	83.5 [77.3–87]	88 [86–90.5]	< 0.0001*
ODI	7.4 [4.0–11.8]	2.0 [1.3–3.3]	< 0.0001*
Hypoxic burden, (%min)/h	13.3 [4.3–33.5]	2.2 [0.92–8.6]	< 0.0001*

Desaturation with the intermittent pattern consists of I, P + I, S + I and S + P + I. Desaturation without the intermittent pattern consists of S, P and S + P. S: Sustained, P: Periodic, I: Intermittent

PM-AHI, CT90%, Lowest SpO_2_, ODI and Hypoxic burden were obtained from a pulse oximeter performed during night

The data are presented as median [interquartile range]. *P < 0.05

*BMI* body mass index, *FVC* forced vital capacity, *FEV*_*1*_ forced expiratory volume in 1 s, *PM-AHI* apnoea-hypopnea index from portable monitor, *CT90* cumulative percentage time at SpO_2_ below 90%

The patients with the sustained pattern had a significantly higher CT90 than those without the sustained pattern (16.4, IQR: 1.9–37.1 vs. 2.1, IQR: 0.35–6.3). On the other hand, PM-AHI was significantly lower in the patients with the sustained pattern (8.4, IQR: 4.3–18.2 vs. 19.2, IQR: 12.1–28.7) (Table 3).

**Table 3:** Association of the sustained pattern with clinical data

	Desaturation with sustained pattern N = 20	Desaturation without sustained pattern N = 108	P value
Male/female	17/3	99/9	0.399
Age, years	71.5 [65.8–76]	71 [68–75]	0.870
BMI	23.4 [21.4–26.2]	22.6 [20.4–24.3]	0.288
Daytime SpO_2,_ %	96 [95–97]	97 [96–98]	0.016*
Smoking history, pack-years	60 [50–81.5]	50 [40–75]	0.114
%FVC, %	96.4 [87.5–110.9]	89.7 [77.4–98.1]	0.031*
%FEV_1_, %	68.8 [16.3–79.7]	62.1 [50.3–76.5]	0.441
FEV_1_/FVC, %	58.2 [45.2–66.2]	60.6 [50.5–66.1]	0.419
PM-AHI	8.4 [4.3–18.2]	19.2 [12.1–28.7]	0.003*
CT90, %	16.4 [1.9–37.1]	2.1 [0.35–6.3]	0.002*
Lowest SpO_2_, %	85 [80–87.5]	84 [78–87]	0.935
ODI	7.8 [4.6–11.9]	4.4 [2.4–8.6]	0.046*
Hypoxic burden, (%min)/h	46.8 [26.3–91.9]	5.9 [2.4–17.1]	< 0.0001*

Desaturation with the sustained pattern consists of S, S + P, S + I and S + P + I. Desaturation without the sustained pattern consists of I, P and P + I. S: Sustained, P: Periodic, I: Intermittent

PM-AHI, CT90%, Lowest SpO_2_, ODI and Hypoxic burden were obtained from a pulse oximeter performed during night

The data are presented as median [interquartile range]. *P < 0.05

*BMI* body mass index, *FVC* forced vital capacity, *FEV*_*1*_ forced expiratory volume in 1 s, *PM-AHI* apnoea-hypopnea index from portable monitor, *CT90* cumulative percentage time at SpO_2_ below 90%

Comparisons between the patients with and without the periodic pattern revealed that there were no significant differences in the PM-AHI. %FEV_1_ was significantly lower in the patients with the periodic pattern (59.8, IQR: 50–66.1 vs. 73.9, IQR: 59.9–81.3) (Table 4).

**Table 4:** Association of the periodic pattern with clinical data

	Desaturation with periodic pattern N = 104	Desaturation without periodic pattern N = 24	P value
Male/female	95/9	21/3	0.696
Age, years	72 [68–75.8]	68.5 [64.3–75]	0.111
BMI	23.0 [20.7–25.2]	21.5 [20.3–23.6]	0.113
Daytime SpO_2,_ %	97 [96–98]	98 [97–98]	0.003*
Smoking history, pack-years	50.5 [40–79.1]	50 [33.8–70]	0.716
%FVC, %	89.4 [76.45–97.4]	98.1 [87.2–107.6]	0.008*
%FEV_1_, %	59.8 [50–66.1]	73.9 [59.9–81.3]	0.039*
FEV_1_/FVC, %	58.2 [45.2–66.2]	61.7 [52.3–66.1]	0.500
PM-AHI	18.4 [10.5–29.0]	16.3 [10.4–22.6]	0.468
CT90, %	6.1 [2.8–14.8]	7.6 [2.8–11.5]	0.738
Lowest SpO_2_, %	83 [79–86]	88 [85.5–90]	< 0.0001*
ODI	7.5 [4.4–11.8]	2.0 [1.4–3.2]	< 0.0001*
Hypoxic burden, (%min)/h	16.8 [6.5–37.2]	1.6 [0.90–3.0]	< 0.0001*

Desaturation with the periodic pattern consists of P, P + I, S + P and S + P + I. Desaturation without the periodic pattern consists of I, S and S + I. S: Sustained, P: Periodic, I: Intermittent

PM-AHI, CT90%, Lowest SpO_2_, ODI and Hypoxic burden were obtained from a pulse oximeter performed during night

The data are presented as median [interquartile range]. *P < 0.05

*BMI* body mass index, *FVC* forced vital capacity, *FEV*_*1*_ forced expiratory volume in 1 s, *PM-AHI* apnoea-hypopnea index from portable monitor, *CT90* cumulative percentage time at SpO_2_ below 90%

Contrary to the hypothesis, the strength of the correlation between the desaturation pattern and the index of respiratory function was low.

## Discussion

In the present study, we investigated nocturnal desaturation in patients with COPD who did not have daytime hypoxemia with an automatic analysis algorithm we previously reported [7].

Even in patients with COPD without daytime desaturation, nocturnal desaturation was common. Sleep affects breathing in various ways, such as through a decrease in ventilator responses to both hypoxia and hypercapnia, a decrease in tidal volume due to the diminished tone and activity of the accessory muscles of respiration, and an increase in upper airway resistance [14, 15].

Although these changes also occur in healthy subjects in an unknown manner [16], the effect of these changes on PaO_2_ can be negligible because of the high oxygen reserve in the lung. On the other hand, patients with COPD have pathological changes such as airflow limitation, respiratory muscle fatigue, hyperinflation of the lungs, and ventilation/perfusion ratio mismatch caused by the destruction of the pulmonary capillary beds [17], which can lead to lower baseline PaO_2_. In patients whose daytime PaO_2_ levels are on the steep portion of the oxyhaemoglobin dissociation curve, even small physiological changes in respiration can lead to greater desaturation [17, 18]. These might be the reasons many patients with COPD have nocturnal desaturation.

In the S + P + I group, there was no trend in %FEV_1_. In other words, it is possible that patients in this group might be exposed to severe hypoxemia regardless of the severity of COPD. Even in patients with COPD without daytime hypoxemia, it has been reported that desaturations can occur due to complications such as obstructive sleep apnoea (OSA) and rapid eye movement (REM) sleep-related hypoventilation [19, 20]. It is suggested that there is a population exposed to severe hypoxemia due to overlap of pathophysiologies, such as apnoea and hypoventilation.

In this study, PM-AHI was significantly higher in desaturation with the intermittent pattern. However, this was not observed in the comparison of patients with and without the sustained or periodic pattern. This might indicate a correlation between the intermittent pattern and PM-AHI. So, we assume this intermittent pattern reflects SAS. Moreover, it is suggested that 99.1% of patients in this pattern were suffering from COPD and SAS.

In each desaturation group, the results of PM-AHI and ODI were not necessarily similar. Because PM-AHI needs to evaluate with both airflow and 3% desaturation, whereas ODI is evaluated only with oxygen desaturation. Assumingly, this difference occurred the dissociation between PM-AHI and ODI results.

In intermittent pattern, the hypoxic burden may be more suitable than CT90 for evaluation the impacts of hypoxic exposure, because it evaluates not only duration of desaturation but also the depth of desaturation [13]. And it is also expected to be applied to other waveforms.

In our previous study, among patients with COPD receiving HOT, the lower %FEV_1_ was, the less desaturation with an intermittent pattern [7]. However, in this study, there was no significant difference. There is growing evidence that lung hyperinflation associated with emphysema reduces the likelihood of OSA [21]. It might be speculated that patients with mild to moderate COPD have mild lung hyperinflation, leading to few effects on desaturation with the intermittent pattern.

In this study, we revealed that there were many overlaps of patients with COPD and intermittent pattern. This made it difficult to evaluate periodic and sustained patterns independently from intermittent pattern. However, we believe it is important to find sleep breathing disorder in COPD is not a single pathophysiology, but often complex.

From the results of the comparison based on the sustained pattern, it could be suggested that patients with the sustained pattern had greater hypoxemic exposure. The characteristic of this sustained pattern is that the SpO_2_ does not recover for a long time, so it is a plausible result. Although there have been reports on the impacts of sustained hypoxic load during sleep [22, 23], the pathophysiology of sustained nocturnal desaturation is not clear. These results suggested that at least this pattern was not involved in apnoea and hypopnea. It has been reported that sustained hypoxemia impairs the arousal response [24, 25], and this arousal response might be involved in sustained desaturation. Nocturnal hypoxemia is generally defined as a SpO_2_ ≤ 88% for more than 5 min [26], among them, there may be a population of patients that we defined as having the sustained pattern.

We assume that the clinical condition of the periodic pattern is REM sleep-related hypoventilation which correlated with elevated PaCO_2_, COPD exacerbation, pulmonary hypertension [20] and poor prognosis [27]. Previous study showed that REM sleep-related hypoventilation is seen in patients with severe COPD and type II respiratory failure [20, 28]. Our result in periodic group is consistent with previous reports in the point that %FEV_1_ was lower in the periodic pattern group. During REM sleep, there are marked diminished in the activity of the accessory muscles of respiration and decrease in ventilator responses in the respiratory center, which can easily lead to alveolar hypoventilation. Based on such speculation, desaturation due to these changes would be more likely to occur in patients with more severe COPD whose pulmonary dysfunction is more severe. On the other hand, Kitajima et al. suggested that NPPV therapy decrease daytime PaCO_2_ and COPD exacerbation frequency, and may improve PH in patients with REM sleep-related hypoventilation [20]. NPPV therapy is also expected to improve prognosis of these of these patients. Therefore we believed that detecting the periodic pattern is clinically important. Although our study observation found no significant association with clinical specific parameters, further studies associating sleep studies and waveform patterns are needed to clarify the clinical utility.

In this study, we evaluated the nocturnal SpO_2_ waveforms and found them to be very diverse. We also classified these waveforms and clarified some of the pathogenesis and the patient backgrounds of each waveform. This might be useful because it helps clarify the pathogenesis of sleep disorders, and warrants further clinical study to lead to provide more appropriate treatment in COPD patients.

The limitations of the present study are the lack of data on polysomnography and overnight transcutaneous carbon dioxide tension. In this study, we used a type 4 portable long-term recordable pulse oximeter to measure SpO_2_ and nasal airflow. According to the classification of the American Sleep Disorder Association, type 4 is determined by the continuous measurement of single or dual bioparameters, for example, oxygen saturation or airflow [29]. This type 4 portable monitor tends to underestimate PM-AHI because the PM-AHI index is defined as the number of apnoeas and hypopneas divided by the total measured time, which is longer than the total sleep time, and the PM-AHI index does not include hypopnea associated with arousal.

## Conclusions

We found that even in patients with COPD without daytime desaturation, nocturnal desaturation was common. We found that the algorithm, which was developed based on data from patients receiving HOT, could be adapted to some extent to patients with COPD, and it captured aspects of the pathogenesis of each desaturation pattern in this study.

## Data Availability

The datasets used and/or analysed during the current study are available from the corresponding author on reasonable request.

## Abbreviations

COPD: Chronic obstructive pulmonary disease
GOLD: Global Initiative for Chronic Obstructive Disease
HOT: Home oxygen therapy
PET: Pulmonary function test
FEV_1_: Forced expiratory volume in 1 s
PM-AHI: Apnoea-hypopnea index from portable monitor
CT90: Cumulative percentage time at SpO_2_ below 90%
ODI: Oxygen desaturation index
IQR: Interquartile range
%FEV_1_: Percent predicted forced expiratory volume in 1 s
OSA: Obstructive sleep apnoea
REM: Rapid eye movement
